# Comparative pharmacokinetics of chlorogenic acid in beagles after oral administrations of single compound, the extracts of *Lonicera japanica*, and the mixture of chlorogenic acid, baicalin, and *Forsythia suspense*

**DOI:** 10.1080/13880209.2017.1296002

**Published:** 2017-03-05

**Authors:** Rong Liu, Ke Lai, Yu Xiao, Jing Ren

**Affiliations:** a School of Medicine and Nursing, Chengdu University, Chengdu, China;; b Antibiotics Research and Re-evaluation Key Laboratory of Sichuan Province, Sichuan Industrial Institute of Antibiotics, Chengdu University, Chengdu, China

**Keywords:** Oral absorption, concomitant compounds, pharmacokinetic parameters, accumulative fraction excretion

## Abstract

**Context:** Chlorogenic acid (ChA) is the major compound in Shuang-Huang-Lian (SHL), which is mainly composed of ChA, baicalin, and *Forsythia suspense* Thunb Vahl.

**Objective:** The effects of co-existing compounds in SHL and *Lonicera japanica* Thunb on the absorption of ChA was investigated.

**Materials and methods:** According to 3 × 3 Latin-square test, ChA alone, the extracts of *Lonicera japanica*, or the mixture of ChA, baicalin and *Forsythia suspense* (ChA effective doses is 60 mg/kg) was separately given to six beagles for seven days. The oral pharmacokinetic parameters of ChA in plasma, urine and faeces were quantified by HPLC/UV and analyzed.

**Results:** The pharmacokinetic parameters of ChA alone, the extracts of *Lonicera japanica*, and the mixture of ChA, baicalin, and *Forsythia suspense* were as followed: *C*_max_ (2.350 ± 0.483, 1.655 ± 0.576, 2.332 ± 0.606 μg/mL), AUC_0-∞_ (6.324 ± 1.853, 4.216 ± 1.886, 6.074 ± 1.473 μg·h/mL), *t*_1/2_ (0.911 ± 0.187, 1.204 ± 0.309, 1.094 ± 0.193 h), and *T*_max_ (1.861 ± 0.499, 1.000 ± 0.459, 1.833 ± 0.279 h). Accumulative fraction excretion of ChA in urine were 0.73 ± 0.55, 1.25 ± 1.23, 1.05 ± 0.96%, while that in faeces were 0.68 ± 0.94, 0.19 ± 0.40, and 1.76 ± 3.57%.

**Discussion and conclusion:** Co-existing compounds in SHL have no effect on the absorption of ChA, while the concomitant compounds in *Lonicera japanica* could decrease that of ChA. ChA in Beagles might have high biological transformation.

## Introduction

In recent years, traditional Chinese medicines (TCM) have gained worldwide popularity for their complementary therapeutic effects to the western drugs, their various pharmacological effects and low side effects (Normile 2003; Xue & Roy 2003). As a commonly used traditional Chinese medicine, SHL is highly prized in TCM practice for its treatment of acute upper respiratory tract infection in clinic. SHL is mainly composed of three herbs: *Lonicera japanica* Thunb, *Scutellaria baicalensis* Georgi and *Forsythia suspense* Thunb Vahl. ChA is a major phenolic compound (no less than 1.5%) and therapeutic ingredient existed in *Lonicera japanica* of SHL. Furthermore, ChA shows a wide range of biological activities, such as killing *Escherichia coli* by hypochlorous acid (Kono et al. 1995), inhibiting hepatic glucose 6-phosphatase (Arion et al. 1997), preventing oxidation (Salvi et al. 2001), and lipid peroxidation (Ohnishi et al. 1994). In previous reports, in order to clarify the destiny of ChA *in vivo*, several papers have described pharmacokinetic studies of ChA after oral administration (Gao et al. 2007; Ren et al. 2007). However, information available was mainly about pharmacokinetics of ChA after oral administration of *Lonicera japanica* (Zhou et al. 2014), or/and SHL (Gao et al. 2014). Still, there has been little research on other compounds in TCM how to influence on the absorption of effective ingredients.

In this work, the pharmacokinetic properties in plasma, urine and feces of ChA were quantified by the HPLC/UV method (Chang et al. 2001; Gao et al. 2007). Based on the oral pharmacokinetics of ChA, the differences of ChA in the forms of single compound, the extracts of *Lonicera japanica* and the mixture of ChA, baicalin and *Forsythia suspense* were compared via oral in beagles in order to reveal the effects of co-existing compounds in SHL and *Lonicera japanica* on the absorption of ChA. So, the research results have important significance to reveal the mechanism of the prescription of ChA.

## Materials and methods

### Reagents and chemicals

ChA and internal standard puerarin were supplied by National Institute for the Control of Pharmaceutical and Biological Products (NICPBP, Beijing, China). Extracts of *Lonicera japanica* (containing 99.8% ChA) were gift samples from Chenguang Research Institute of Chemical Engineering (Chendu, China). Extracts of *Lonicera japanica* (containing 37.2% ChA) and extracts of *Forsythia suspense* Vahl (containing about 1.4% forsythin and 9.0% forsythoside) were bought from Sichuan Mingbo Pharmaceutical Co., LTD (Chengdu, China). Extracts of *Scutellaria baicalensis* (containing 97.0% baicalin) were bought from Sichuan Sunnyhope Pharmaceutical Co., LTD (Chendu, China). Methanol and acetonitrile (HPLC grade) were purchased from Dikma Technologies (Beijing, China). Phosphoric acid, acetic acid, trichloroacetic acid, disodium hydrogen phosphate were of analytical grade and obtained from the Chengdu Reagent Company (Chengdu, China). Water was prepared by an ultra-pure water system (UPA, Chongqing, China). All other chemicals used in the study were of analytical grade at least.

### Animals, drug administration, and sampling

The Sichuan University animal ethical experimentation committee, according to the requirement of the National Act on the Use of Experimental Animal (People’s Republic of China), approved all procedures of the animal study (number: GPT 5-1). Beagles weighting 9.0–11.2 kg (Table 1) (half male and half female) were obtained from the Laboratory Animal Center at West China School of Pharmacy, Sichuan University.

**Table 1.** Sex and weight of beagles.

No.	A	B	C	D	E	F
Sex	♀	♂	♀	♂	♂	♀
Weight (kg)	9.6	11.2	9.0	11.0	9.2	10.4

The beagles in this study were subjected to recirculating perfusion according to 3 × 3 Latin square test design. Six beagles were randomly assigned to six groups and had free access to water and food by the following seven days. The beagles fasted for 12 h with free access to water before dosing. On the day of experiment, the animal was separately dosed with the ChA alone, the extracts of *Lonicera japanica* or the mixture of ChA, baicalin, and *Forsythia suspense* via the oral route (Table 2). According to the clinical dose for human, the effective dose 60 mg/kg ChA was selected for the beagles after dose conversion. Serial blood samples (about 0.5 mL) were collected at 0, 10, 20, 30, 45, 60, 80, 100, 120, 150, 180, 240, and 320 min; serial urine samples were collected at 0 h, 0–4 h, 4–8 h, 8–12 h, and 12–24 h with the precisely measured volume; serial faeces samples were collected at 0 h, 0–12 h, and 12–24 h. The procedures of the second and third administrations were the same as mentioned above.

**Table 2.** Administration order.

Group						
circle	A	B	C	D	E	F
1	T1^b^	R^a^	T2^c^	T1^b^	T2^c^	R^a^
2	R^a^	T2^c^	T1^b^	T2^c^	R^a^	T1^b^
3	T2^c^	T1^b^	R^a^	R^a^	T1^b^	T2^c^

a R-reference: 60 mg/kg ChA contained in extracts of *Lonicerae japonicae flos* (containing 99.8% ChA).

b T1-test 1:60 mg/kg ChA contained in extracts of *Lonicerae japonicae flos* (containing 99.8% ChA) + 12 mg/kg extracts of *Forsythia suspense vahl* (containing 1.4% forsythin and 9.0% forsythoside)+6 mg/kg extracts of *Scutellaria baicalensis georgi* (containing 97% baicalin).

c T2-test 2: 60 mg/kg ChA contained in extracts of *Lonicerae japonicae flos* (containing 37.23% ChA).

Blood samples were taken into heparinized microcentrifuge tubes, and centrifuged immediately at 11,000 rpm for 5 min. The plasma layers were separated and stored in microcentrifuge tubes at −20 °C until the analysis. Five millilitres of urine samples and all of faeces samples were stored at −70 °C until the analysis.

### Calibration standards and quality control samples

To prepare a series of standard plasma samples, 100 μL of ChA standard solution with different concentrations and 100 μL of internal standard solution (puerarin, 100 μg/mL) were separately added into 0.5 mL of blank plasma. And then the mixture was vortex-mixed thoroughly to get the final standard solution with the concentrations of 0.404, 0.808, 1.615, 3.230, 6.460, 12.920, and 25.840 μg/mL. In the same manner, the quality control (QC) samples were also prepared at the concentrations of 0.404, 0.808, 6.460 and 25.840 μg/mL.

To prepare a series of standard urine samples, 50 μL of ChA standard solution with different concentrations was respectively added into 0.5 mL of blank urine, followed by vortex mixture to get final concentrations of 1.292, 6.460, 12.92, 64.60, and 129.2 μg/mL. QC samples were also prepared at concentrations of 1.292, 6.460, 12.92, and 129.2 μg/mL in the same manner.

A series of standard faeces samples and QC samples were prepared by the same method as that of urine samples (concentrations of the standard solution: 0.646, 1.292, 12.92, 64.60, and 129.2 μg/mL; concentrations of QC samples: 0.646, 1.292, 12.92 and 129.2 μg/mL).

All the above solutions were stored at 4 °C before use for no longer than one month.

### Sample preparations

To 0.5 mL of plasma sample, 100 μL of internal standard solution, 100 μL of methanol:0.2% phosphoric acid (20:80, v/v) and 0.5 mL of 10% solution of trichloroacetic were successively added into a centrifuge tube. Subsequently, the mixture was vortex-mixed for 2 min (vortex WH-3, Anting Scientific Instrument, Shanghai, China), and then centrifuged at 12,000 rpm for 10 min (Anke TGL-16C centrifuge, Anting Scientific Instrument, Shanghai, China). Twenty microliters of supernatant was manually injected into the HPLC for analysis.

To 0.5 mL of urine samples or faeces homogenates, 50 μL of methanol:0.2% phosphoric acid (20:80, v/v) and 0.5 mL of 10% solution of trichloroacetic were added into a centrifuge tube, and other procedures were the same as that of plasma sample.

### Analytical procedure

The HPLC apparatus included a Series III Pump, a Model 500 UV/VIS detector and a 7725i injection valve. Data collection and integration were accomplished by Shimadzu EZ Start 7.1.1 program software.

Chromatographic separation was performed on reversed-phase Scienhome Kromasil C_18_ column (250 mm × 4.6 mm, 5 μm) (Dikma technologies, Beijing, China). ChA in plasma was separated by mobile phase consisted of methanol, acetonitrile, and disodium hydrogen phosphate (5 mM, pH 3.0 adjusted by acetic acid) (15:20:170, v/v/v); ChA in urine or faeces was separated by mobile phase consisted of methanol, acetonitrile, and disodium hydrogen phosphate with a different volume ratio of 6:8:88. Separation was carried out isocratically at 35 °C with a flow rate of 1.0 mL/min, and UV detection was performed at 322 nm (Chang et al. 2001; Gao et al. 2007).

### Precision and accuracy

In order to determine the intra- and inter-day accuracy and precision, six replications of all QC samples were performed. Each concentration of samples was calculated according to the calibration curve. The intra- and inter-day mean accuracy was determined by the ratio of calculated concentration and nominal concentration within 10%, and the precision was evaluated by relative standard derivative (RSD) within 15%.

### Extraction recovery and stability of ChA samples

The recovery of ChA was assessed by the six replications of QC samples in plasma. Firstly, plasma samples containing ChA or puerarin were post-processed by the following steps. Concretely, 100 μL of internal standards, 0.5 mL of 10% solution of trichloroacetic, and 100 μL of methanol:0.2% phosphoric acid (20:80, v/v) were added into 0.5 mL of plasma samples and then vortexed for 2 min. Secondly, solution was centrifuged, and 20 μL of supernatant was injected into HPLC. Finally, the peak area of ChA (A_1_) or puerarin (A_2_) in plasma samples was separately obtained. The control group, water, was also post-processed and detected by the same method as above, and then the peak areas of ChA (A_1_′) and puerarin (A_2_′) were acquired. The extraction recovery was calculated by the following formula: ChA in plasma samples (%) = A_1_/A_1_′; puerarin in plasma samples (%) = A_2_/A_2_′.

Six replications of QC samples in plasma, urine and faeces were chosen to investigate the stability of ChA. The short-term stability of ChA was evaluated by keeping QC samples at room temperature for 6 h. The post-preparative stability was conducted by putting QC samples after operation at 4 °C for 8 h. Freeze-thaw stability was determined by separately assessing QC samples in plasma after freeze at −20 °C, and those in urine and faeces after freeze at −70 °C, and then thaw cycles at room temperature three times. The long-term stability was determined by placing QC samples in plasma at −20 °C and those in urine and faeces at −70 °C.

### Data analysis

Concentrations were estimated for ChA in plasma after intragastric administration, which was based on the calibration curve relating peak area of ChA/puerarin to ChA concentration spiked in samples. And ChA concentrations in urine and faeces were estimated by the calibration curve, which were obtained by plotting peak area of ChA versus ChA concentrations spiked in samples. In addition, calculation was performed by SPSS (version 10.0, IBM Corp., Armonk, NY).

Pharmacokinetic data were subsequently processed by the Drug and Statistics ver 1.0 Program (DAS, Anhui, China), such as area under the plasma concentration–time curve (AUC) and half-life (*t*_1/2_). *C*_max_ and *T*_max_ were obtained from the experimental data. What’s more, accumulative excretion of ChA in urine was accessed by variance analysis, and that in faeces was accessed by rank sum test. And a *p*-value >0.05 was considered as no significant.

## Results

### Specificity

Chromatograms of ChA in beagle plasma, urine and faeces showed that the retention times of ChA and internal standard puerarin were approximately 7.5 and 11.0–13.0 min, respectively, with complete baseline resolution between peaks of interest. No interfering peaks were observed at the retention time of ChA and internal standard in all the conditions.

### Precision and accuracy

The intra- and inter-day precisions were calculated by replicated assays of ChA in plasma, urine and faeces samples, and the data were shown in Tables 3–5. Precision and accuracy studies indicated that the developed HPLC method was reproducible and accurate.

**Table 3.** Accuracy and precision of ChA in plasma samples.

Added concentration (μg/mL)	Mean measured concentration (μg/mL)	Accuracy (%)	RSD (%)
Intraday (*n* = 6)			
25.84	25.69	99.4	1.44
6.46	6.36	98.5	1.64
0.808	0.814	100.7	4.33
0.404	0.412	102.0	8.91
Interday (*n* = 24)			
25.84	25.815	99.9	2.47
6.46	6.55	101.4	3.00
0.808	0.793	98.1	5.26
0.404	0.412	102.0	8.96

**Table 4.** Accuracy and precision of ChA in urine samples.

Added concentration (μg/mL)	Mean measured concentration (μg/mL)	Accuracy (%)	RSD (%)
Intraday (*n* = 6)			
129.2	126.8	98.1	0.69
12.92	12.34	95.5	2.44
6.46	6.216	96.2	5.72
1.292	1.321	102.2	4.10
Interday (*n* = 24)			
129.2	135.0	104.5	4.53
12.92	12.21	94.5	2.42
6.46	5.97	92.4	5.35
1.292	1.361	105.3	4.87

**Table 5.** Accuracy and precision of ChA in faeces samples.

Added concentration (μg/mL)	Mean measured concentration (μg/mL)	Accuracy (%)	RSD (%)
Intraday (*n* = 6)			
129.2	128.4	99.4	0.23
12.92	13.17	101.9	2.1
1.292	1.276	98.8	3.75
0.646	0.677	104.8	2.94
Interday (*n* = 24)			
129.2	128.7	99.6	1.35
12.92	12.97	100.4	3.59
1.292	1.206	93.3	6.48
0.646	0.665	102.9	6.66

### Linearity, LLOQ, and LOD

All of the correlation coefficients of calibration curve in plasma, urine and faeces samples were larger than 0.99, suggesting a good linearity within the different concentration ranges of 0.404–25.840 μg/mL of ChA in plasma, 1.292–129.2 μg/mL of ChA in urine and 0.646–129.2 μg/mL of ChA in faeces.

The limit of detection (LOD) for this method defined as a signal-to-noise ratio of 3:1 was 0.053 μg/mL. The lower limit of quantitation (LLOQ) defined as the lowest drug concentration, which can be determined with an intra-day relative standard deviation ≤20%, was estimated as 0.105 μg/mL.

### Extraction recovery and stability of ChA samples

The result of stability (Tables 6–8) indicated that at 4 °C, ChA was stable for at least 4 h in processed plasma and at least 8 h in processed urine or faeces. At room temperature, it was stable for at least 3 h in plasma and 6 h in urine or faeces. At −20 °C, it in plasma was stable for at least one month, while at −70 °C, for at least 20 d in urine and 7 d in faeces. Moreover, there was no significant degradation of ChA after three freeze and thaw cycles, which indicated that ChA was stable enough for injection onto HPLC system.

**Table 6.** Stability of ChA in beagle plasma under various storage conditions (Mean ± SD, *n* = 3).

Nominal conc. (μg/mL)	4 °C/4 h (extracted sample)	Room temp./3 h	−20 °C/3 freeze/thaw cycles	−20 °C/1 mon
25.840	102.75 ± 0.35	94.97 ± 1.34	98.69 ± 2.76	114.28 ± 0.99
6.460	103.64 ± 1.78	93.22 ± 1.18	97.16 ± 4.62	103.12 ± 0.34
0.808	100.50 ± 8.03	96.48 ± 0.16	98.88 ± 4.68	107.79 ± 7.90

**Table 7.** Stability of ChA in beagle urine under various storage conditions (Mean ± SD, *n* = 3).

Nominal conc. (μg/mL)	4 °C/8 h (extracted sample)	Room temp./6 h	−70 °C/3 freeze/thaw cycles	−70 °C/20 d
129.2	99.87 ± 0.38	99.84 ± 0.13	102.69 ± 0.69	96.78 ± 1.06
12.92	101.27 ± 0.92	100.28 ± 1.60	103.87 ± 1.25	100.65 ± 1.24
6.46	99.80 ± 0.78	102.17 ± 2.00	109.80 ± 14.79	101.32 ± 3.94

**Table 8.** Stability of ChA in beagle faeces under various storage conditions (Mean ± SD, *n* = 3).

Nominal conc. (μg/mL)	4 °C/8 h (extracted sample)	Room temp./6 h	−70 °C/3 freeze/thaw cycles	−70 °C/7 d
129.2	101.11 ± 1.22	87.18 ± 1.50	96.43 ± 4.06	99.88 ± 1.69
12.92	101.04 ± 1.16	86.17 ± 2.13	96.18 ± 1.07	98.62 ± 1.34
1.292	91.37 ± 1.93	105.12 ± 11.35	100.41 ± 2.78	101.46 ± 3.21

The mean extraction efficiency was 88.57% for ChA, and 89.81% for the internal standard (*n* = 6) from plasma.

### Pharmacokinetic analysis

**Figure 1.**
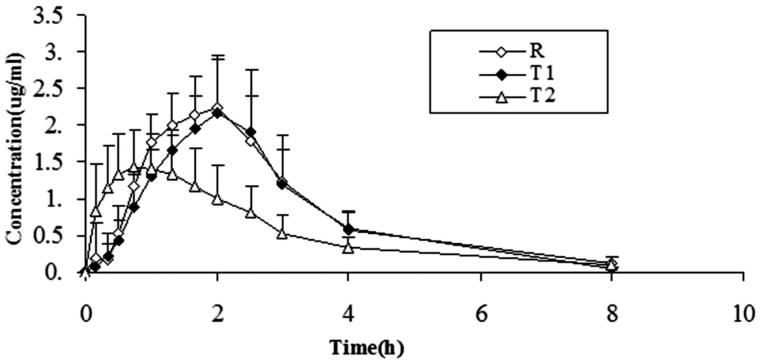
Concentration–time curve in beagle plasma.

Mean plasma concentration-time profile and the corresponding pharmacokinetic parameters of ChA after oral administration was separately shown in Figure 1 and Table 9. Compared with the ChA in plasma of beagles dosed with extracts of *Lonicera japanica* (containing 37.23% ChA), that in plasma of beagles dosed with ChA alone showed higher *C*_max_ (2.350 ± 0.483 μg/mL), AUC_0-t_ (5.591 ± 1.406 μg·h/mL), AUC_0-∞_ (6.324 ± 1.853 μg·h/mL), *t*_1/2_ (0.911 ± 0.187 h), and T_max_ (1.861 ± 0.499 h). Nevertheless, in beagles co-dosed with ChA, baicalin, and *Forsythia suspense*, the ChA of plasma showed the similar *C*_max_ (2.332 ± 0.606 versus 2.350 ± 0.483 μg/mL), AUC_0-t_ (5.325 ± 1.341 versus 5.591 ± 1.406 μg·h/mL), AUC_0-∞_ (6.074 ± 1.473 versus 6.324 ± 1.853 μg·h/mL), *t*_1/2_ (1.094 ± 0.193 versus 0.911 ± 0.187 h), and *T*_max_ (1.833 ± 0.279 versus 1.861 ± 0.499) with that of beagles dosed with ChA alone.

**Table 9.** Pharmacokinetics parameters for ChA (*n* = 6).

	R	T1	T2
*t*_1/2_ (h)	0.911 ± 0.187	1.094 ± 0.193	1.204 ± 0.309^a^
*C*_max_ (μg/mL)	2.350 ± 0.483	2.332 ± 0.606	1.655 ± 0.576^a^
*T*_max_ (h)	1.861 ± 0.499	1.833 ± 0.279	1.000 ± 0.459^a^
AUC_0-t_ (μg·h/mL)	5.591 ± 1.406	5.325 ± 1.341	3.792 ± 1.794^a^
AUC_0-∞_ (μg·h/mL)	6.324 ± 1.853	6.074 ± 1.473	4.216 ± 1.886^a^
*F* (%)		96.0	66.7

a *p* < 0.05, compared with R, T2 has statistical significance.

Accumulative fraction excretion of ChA in urine of R, T1, or T2 groups was, respectively, 0.73%, 1.25%, and 1.05%, as well as that of ChA in faeces was, respectively, 0.68%, 0.19%, and 1.76%, which suggested that ChA without being absorbed was almost metabolized in gastrointestinal tract of beagles.

## Discussion

The pharmacokinetic parameters had remarkable differences between ChA alone and extracts of *Lonicera japanica* (containing 37.23% ChA) by oral in beagles. In the extracts of *Lonicera japanica*, the other concomitant compounds also existed except for ChA, and they might have the same uptake pathway as ChA. *C*_max_ of ChA decreased in extracts of *Lonicera japanica* (containing 37.23% ChA) and relative bioavailability of it was only 66.7%, suggesting that concomitant compounds in extracts of *Lonicera japanica* interfered with the absorption of ChA. Therefore, it’s best to choose the extracts with higher content of ChA for preparation.

To the mixture of ChA, *Scutellaria baicalensis* (containing 97% baicalin) and *Forsythia suspense* (containing 1.4% forsythin and 9.0% forsythoside), there was the same absorption and elimination profile of ChA as those of ChA alone. Thereby, co-existing compounds in SHL had no effects on the absorption of ChA. In TCM, *Scutellaria baicalensis* and *Forsythia suspense* are usually combined with ChA. Moreover, combination preparation has better clinic efficacy than ChA alone. However, whether ChA effects on the in vivo absorption of *Scutellaria baicalensis* and *Forsythia suspense* is not clear and still need further research.

In addition, in R, T1, or T2 groups, accumulative fraction excretions of ChA were very low in urine or faeces, which indicated that ChA could be bio-transformed before exerted from body. ChA is vulnerable to oxidation, because it is a selective hydroxyl free radical scavenger and oxygen free radical scavenger. Therefore, ChA in Beagle dogs might have high biological transformation, which led to very few prototype drugs detected.

## Conclusion

In conclusion, other co-existing compounds in SHL had no effects on the absorption of ChA, but the concomitant compounds in extracts of *Lonicera japanica* made the absorption ChA less. Therefore, high-purity ChA should be used for preparation. In addition, there was no remarkable difference of accumulative fraction excretions of ChA in urine or faeces of beagles dosed in R, T1, or T2 groups. Furthermore, a trace amount of ChA in the parent form was detected in the urine and faeces. Further studies are ongoing in our laboratory in order to investigate the *in vivo* process of ChA with co-existing components.
